# Effect of stress coping ability and working hours on burnout among residents

**DOI:** 10.1186/s12909-020-02134-0

**Published:** 2020-07-13

**Authors:** Saori Kijima, Kazuya Tomihara, Masami Tagawa

**Affiliations:** 1 Center for Innovation in Medical and Dental Education, Graduate School of Medical and Dental Sciences, Kagoshima University, 8-35-1 Sakuragaoka, Kagoshima, 890-8544 Japan; 2 Osumi Kanoya Hospital, 6081-1 Shinkawacho, Kanoya, Kagoshima, 893-0015 Japan; 3 Department of Psychology, Faculty of Law, Economics, and Humanities, Kagoshima University, 1-21-30 Korimoto, Kagoshima, 890–0065 Japan

**Keywords:** Burnout, Maslach burnout inventory, Sense of coherence, Resident, Working hours

## Abstract

**Background:**

Burnout among residents leads to interruptions in training and even to exit from programs. Despite the implementation of working hour restrictions in the U.S. in 2013, the high rate of burnout remains a serious problem. Therefore, we analyzed Japanese residents’ burnout, training conditions, and associated factors, especially stress coping ability, which could become an evidence base for creating guidelines of programs and working environments.

**Methods:**

In total, 37 teaching hospitals were randomly selected, and all residents in the third and fifteenth months of a residency program at these hospitals were targeted for this research. We analyzed the residents’ burnout rates, associated factors, and interactions using response data from a self-administered questionnaire consisting of the Japanese versions of the Maslach Burnout Inventory (MBI) and the Sense of Coherence (SOC) scale, as well as items asking about their training environments, gender, and age.

**Results:**

Overall, 48 (49.5%) of 97 residents in 18 teaching hospitals (62 and 35 in the third and fifteenth months, respectively), whose average working hours were 63.3 h per week, were judged as having burnout, among whom, 33 (53.2%) and 15 (42.9%) had burnout in the third and fifteenth months, respectively. Logistic regression analysis indicated that working hours and 10 items on the SOC scale (SOC10) were significant factors of burnout. Two-way analysis of variance revealed that working hours was a significant variable for the MBI-emotional exhaustion score and SOC10 in the third and fifteenth months, respectively. Regarding the MBI-cynicism and professional efficacy scores, the SOC10 was a significant variable in both the third and fifteenth months. In addition, the high SOC group (SOC10 > 45) showed higher personal efficacy under longer working hours.

**Conclusion:**

About half of the Japanese residents were judged as having burnout as early as the third month of training under regulations of working 40 h per week. Individual stress coping ability and working hours were found to be significant factors for burnout. Residents with high stress coping ability exhibited more personal efficacy with more working experiences, which suggests that the SOC scale could be a valuable tool to help foster a suitable training environment.

## Background

Postgraduate residency programs in the United States, Canada, and many other countries now aim to foster medical doctors who possess competencies as professionals. To accomplish this goal, qualified residency programs under governmental or professional regulations and guidelines provide residents with opportunities for essential clinical training as well as necessary support and a proper working environment.

Burnout is defined as a prolonged work-related response to emotional and interpersonal stressors on the job, and has been associated with withdrawal, intention to leave the job, job turnover, loss of productivity, and quality of work [1–3]. Maslach and Jackson developed the Maslach Burnout Inventory (MBI) [1], which is composed of three subscales that evaluate the burnout dimensions of emotional exhaustion, cynicism (depersonalization), and professional efficacy (personal accomplishment). The MBI has been used for evaluating emotional state and judging burnout [3].

Medical and health professionals, among others, might easily fall into burnout because of excessive demand for mental energy in the process of assisting patients [4]. Previous studies using the MBI have reported that 76% of internal medicine residents in the United States in 2001 [5] and 61% of residents in Australia in 2001 [6] were judged as having burnout.

Maslach et al. analyzed the causes of burnout based on interviews with company administrators and workers, and reported that burnout was the result of factors such as social environmental problems, overtime work, a lack of discretionary power, inadequate remuneration, dissatisfaction with work, poor human relations, and the uncontrollability of work [7]. As for residents’ burnout, risk factors and effective interventions have repeatedly been discussed [3]. Several studies have reported that inadequate remuneration [8] and frequent calls [9] and night duty [10] are promoting (or worsening) factors for burnout, and that 80 or fewer hours of work per week [11], counseling [12, 13], the existence of somebody to consult with, such as those in mentoring programs [14, 15], stress-relieving opportunities [16, 17], and mindfulness-based skills programs [18] are preventive (or relieving) factors for burnout.

In addition to the training environment, individual factors such as marriage [19] and childcare [20] have been reported to reduce the factors of burnout. Men have reported significantly more job- and patient-related burnout than women [21], whereas women are more likely than men to report frequently ‘experiencing fatigue’ and ‘burnout from work’ [22]. In addition, Tsele et al. and Cliiers reported that individual stress coping ability affects burnout among health professionals [23, 24].

Antonovsky proposed the concept of salutogenesis, and developed the Sense of Coherence (SOC) scale to evaluate individual stress coping ability [25]. The SOC scale is a self-administered questionnaire composed of the following three subscales: the extent to which a person comprehends the world (comprehensibility), perceives manageability in whatever situation that arises (manageability), and finds meaning in life (meaningfulness). A previous study involving 79 nurses using the SOC scale, MBI, and Beck’s Depression Inventory indicated that people with a low SOC score were at higher risk of burnout and depression [23]. Tartas et al. reported that SOC scores examined prior to medical school admission were significantly correlated with occupational stress and burnout among medical doctors after graduation [26].

In the United States, the Accreditation Council for Graduate Medical Education implemented working hour regulations on residency programs to prevent overwork, resulting in an average of 80 working hours per week [27–29]. Although the frequency of burnout decreased from 36 to 77% to 25–69% [28, 30–33], burnout remains a serious problem. Therefore, in addition to uniform restrictions on working hours, residency programs require evidence-based guidelines for fostering effective working environments.

The aim of the present study was to reveal the frequency of burnout among Japanese residents and the level of emotional exhaustion, cynicism, and professional efficacy, which characterize a burned out person, at different training phases, and to reveal environmental and individual factors related to burnout. Furthermore, this study aimed to explore the influence of individual stress coping ability on burnout and working hours.

## Methods

### Targets and data collection

All postgraduate residency programs in Japan are qualified by the Ministry of Health, Labour and Welfare. It is mandatory for residents to practice internal medicine, general surgery, community medicine, and emergency medicine in a 2-year program. We randomly selected 37 teaching hospitals of various sizes that operate qualified residency programs based on geological distribution (1–6 hospitals/prefecture) from all over Japan.

To collect data at the early and later phases of training, all residents in the third and fifteenth months at these hospitals were targeted for this research.

Next, we created a self-administered questionnaire consisting of the following:

1. Japanese version of the MBI-General Survey (MBI-GS)™ (Mind Garden, Inc. Menlo Park, CA, USA) [34]. The MBI-GS is a scale composed of 16 items rated on a seven-point Likert scale. The Japanese version of the MBI-GS was created and validated by Kitaoka et al. [35]. Kitaoka granted the authors of the present study permission to use the validated Japanese version.
2. Japanese version of the SOC scale. The SOC scale is composed of 13 items rated on a seven-point Likert scale. The Japanese version of the SOC scale was created and validated by Togari et al. [36]. Yamazaki (a coauthor of that study) granted the authors of the present study permission to use the validated Japanese version.
3. Items regarding influential factors in the training environment, including frequency of night duty [10], working hours [11], existence of somebody to consult with [14, 15], having ways to release one’s stress [16, 17], feeling adequately rewarded for one’s own work [8], and work controllability [7], and
4. Personal characteristics such as gender and age.

We distributed the printed questionnaire by mail with an exploratory description of this research and its ethical approval, and collected responses from July to September 2014.

### Data analysis

We confirmed the independence of the MBI and SOC scales by exploratory factor analysis with promax rotation using item scores of both scales, and then excluded the SOC items that were classified into the same factors as the MBI subscale for the subsequent analysis. We confirmed the internal consistencies of the MBI, MBI subscales, and SOC scale using Cronbach’s α coefficient.

The cut-off and abnormal scores for each MBI subscale, which evaluate different features of burnout, as indicated by the MBI-GS were as follows:

- MBI-GS subscale emotional exhaustion (MBI-EX) score: 16 or higher
- MBI-GS subscale cynicism (MBI-CY) score: 11 or higher
- MBI-GS subscale professional efficacy (MBI-PE) score: 23 or lower

Individual burnout judgment varies in the literature, so we adopted the following criteria validated by Schaufeil et al. [37]: an MBI-EX score of 16 or higher and/or an MBI-CY score of 11 or higher. These criteria can discriminate between clinical burned out and not-burned out employees [37], and have been used in previous burnout studies [5, 6].

Next, we analyzed the frequency of burnout calculated with the number of residents judged by these criteria, MBI-EX, MBI-CY, and MBI-PE scores, influential factors related to these scores, and differences in training phases using the *t*-test, chi-squared test, and logistic regression analysis. To assess the effects of stress coping ability on burnout, the respondents were divided into low and high SOC groups using the average SOC scores since a cut-off score for stress coping ability has not been reported. Correlation and two-way analysis of variance was then carried out to analyze the relationship between MBI-EX, MBI-CY, and MBI-PE scores, SOC scores, and working hours. SPSS (version 21; IBM, New York, NY, USA) was used for all data analyses.

## Results

In total, 107 residents (response rate: 28.2%) at 18 teaching hospitals in 11 prefectures responded to the questionnaire. After excluding invalid responses, such as choosing the same options, data from 97 residents (41 residents in hospitals with 500 beds or more, 36 in hospitals with 300–499 beds, and 20 in hospitals with 299 beds or less; 62 in the third month of their residency program and 35 in the fifteenth) were used for the analysis.

Table 1 shows the respondents’ demographic data and working environments. The residents’ average ages in the third and fifteenth months were 26.7 and 27.6 years, respectively, average frequency of night duty was 3.6 times per month, and average number of working hours per day was 11.5, which is equivalent to 63.3 h per week.

**Table 1:** Baseline demographics of the survey respondents

	Third month		Fifteenth month		Total	
	n	(%)	n	(%)	n	(%)
Respondent	62	(100)	35	(100)	97	(100)
Male	37	(59.7)	26	(74.3)	63	(64.9)
Female	25	(40.3)	9	(25.7)	34	(35.1)
	Mean	(SD)	Mean	(SD)	Mean	(SD)
Age (years)	26.7	(3.6)	27.6	(3.5)	27.1	(3.6)
Working environment						
Night duty (days/month)	3.8	(2.2)	3.3	(2.3)	3.6	(2.3)
Working hours (h/day)	11.9	(2.7)	10.9	(1.7)	11.5	(2.4)

n: number of respondents. *SD* standard deviation

### Confirmation of scales

Exploratory factor analysis with promax rotation of the Japanese versions of the MBI and SOC scale using data from the 97 respondents indicated a six-factor structure (Table 2). Factors 1, 2, and 4 had items identical to the MBI-PE, MBI-EX, and MBI-CY detected in the original English version. The three-factor structure of the original version of the SOC scale did not detect these in the present analysis, and two items on the SOC scale were classified into factor 1 (MBI-PE); one item had a loading of over 0.4 for factor 4 (MBI-CY) and factor 6. These three items were excluded, and thus, 10 items of the SOC scale (SOC10) were used for the analysis.

**Table 2:** Promax-rotated pattern/structure coefficients for each factor, extracted communalities (h^2^), and eigenvalues for SOC and MBI factor analysis of scores from 97 residents

Item	Factor						
	1	2	3	4	5	6	h^2^
MBI16	**0.880**	−0.087	− 0.012	0.086	0.090	−0.027	0.757
MBI10	**0.873**	−0.181	− 0.004	0.164	0.041	−0.102	0.735
MBI11	**0.768**	0.038	−0.154	−0.068	− 0.011	0.080	0.552
MBI7	**0.757**	0.079	0.054	−0.052	0.025	0.002	0.639
MBI12	**0.683**	0.137	−0.103	− 0.218	0.056	0.272	0.625
MBI5	**0.561**	0.163	0.076	−0.205	−0.180	− 0.092	0.487
SOC4	0.298	0.162	0.157	−0.070	0.056	0.189	0.209
XSOC10	0.271	−0.198	0.018	−0.061	0.017	−0.202	0.187
MBI2	−0.026	**0.906**	−0.063	−0.089	0.044	0.036	0.774
MBI1	0.013	**0.821**	−0.069	0.001	0.000	0.084	0.686
MBI6	0.047	**0.781**	0.207	0.194	−0.019	0.195	0.619
MBI3	−0.049	**0.746**	−0.009	0.094	−0.009	−0.138	0.728
MBI4	−0.004	**0.645**	−0.054	0.253	0.009	−0.109	0.720
SOC12	−0.027	0.144	**0.783**	−0.245	−0.048	0.109	0.767
SOC9	−0.031	−0.067	**0.769**	0.088	0.078	−0.089	0.575
SOC8	0.118	−0.048	**0.718**	0.007	−0.031	−0.352	0.663
SOC5	−0.239	−0.126	**0.645**	0.069	0.009	0.106	0.427
SOC13	0.001	0.176	**0.624**	−0.194	−0.008	−0.061	0.463
SOC6	0.301	−0.284	**0.421**	0.265	−0.203	0.077	0.375
SOC11	0.095	−0.036	0.365	−0.078	0.212	−0.315	0.370
MBI15	−0.108	0.014	−0.067	**0.787**	0.087	0.035	0.728
MBI8	−0.019	0.246	0.043	**0.762**	0.044	−0.112	0.810
MBI14	−0.049	−0.035	− 0.143	**0.717**	0.018	0.187	0.624
MBI9	−0.001	0.157	0.017	**0.716**	−0.051	−0.142	0.720
MBI13	0.212	0.38	0.010	**0.421**	−0.090	−0.064	0.473
XSOC2	0.147	0.020	−0.153	0.047	**0.815**	−0.133	0.633
XSOC3	−0.122	−0.009	0.162	0.037	**0.735**	0.044	0.604
XSOC1	0.057	0.001	0.264	−0.076	**0.410**	0.080	0.387
XSOC7	0.123	−0.130	−0.048	**− 0.420**	0.005	**0.468**	0.593
Rotated sums of squared loadings	5.375	5.328	5.490	6.320	2.461	1.324	

*MBI* Maslach Burnout Inventory, *SOC* Sense of Coherence scale, *XSOC* reverse coding item of SOC, *MBIx* MBI item number x, *SOCy* SOC item number y

We used the main factor method to extract factors

We defined a high factor loading as ≥0.4. Factor analysis was performed by exploratory analyses

Cronbach’s α coefficients for the MBI-EX, MBI-CY, and MBI-PE were 0.91, 0.88, and 0.89, respectively. Cronbach’s α coefficient for the SOC10 was 0.81.

### MBI-GS subscale scores, burnout frequency, and influential factors

As shown in Table 3, the residents’ mean MBI-EX, −CY, and -PE scores were 15.2, 7.7, and 16.4, respectively.

**Table 3:** Residents’ mean scores for the MBI-GS subscales and SOC10

		Third month (*n* = 62)		Fifteenth month (*n* = 35)		Total (*n* = 97)	
		Mean	SD	Mean	SD	Mean	SD
MBI	EX	16.0	7.0	13.6	7.0	15.2	7.1
	CY	7.4	6.5	8.3	7.5	7.7	6.9
	PE	16.9	8.0	15.4	6.9	16.4	7.6
SOC10		45.0	10.0	45.0	10.5	45.0	10.1

*MBI* Maslach Burnout Inventory, *EX* emotional exhaustion, *CY* cynicism, *PE* professional efficacy; SOC10: scores on the Sense of Coherence scale, excluding three items that were classified into the Maslach Burnout Inventory subscales. n: number of respondents. *SD* standard deviation

Among 97 respondents, 48 (49.5%) were judged as having burnout (Table 4). The frequencies of burnout in the third and fifteenth months were 53.2 and 42.9%, respectively, with no significant difference. The frequencies of burnout among men and women were 49.2 and 50.0%, respectively; gender was not a significant factor.

**Table 4:** Number and percentage of burnout according to the participants’ gender and working environment

	Third month			Fifteenth month			Total		
	Respondents	Burnout		Respondents	Burnout		Respondents	Burnout	
Variable	n	n	(%)	n	n	(%)	n	n	(%)
All	62	33	(53.2)	35	15	(42.9)	97	48	(49.5)
Male	37	22	(59.5)	26	9	(34.6)	63	31	(49.2)
Female	25	11	(44.0)	9	6	(66.7)	34	17	(50.0)
Night duty (days/month)									
≤ 2	15	5	(33.3)	10	6	(60.0)	25	11	(44.0)
≤ 4	23	13	(56.5)	16	8	(50.0)	39	21	(53.8)
≤ 6	17	10	(58.8)	7	1	(14.3)	24	11	(45.8)
> 6	5	4	(80.0)	2	0	(0)	7	4	(57.1)
Working hours (h/day)									
≤ 9	11	4	(36.4)	8	1	(12.5)	19	5	(26.3)
≤ 11	18	8	(44.4)	11	5	(45.5)	29	13	(44.8)
≤ 13	19	10	(52.6)	13	7	(53.8)	32	17	(53.1)
> 13	14	11	(78.6)	3	2	(66.7)	17	13	(76.5)
Existence of somebody to consult with									
Yes	57	30	(52.6)	33	14	(42.4)	90	44	(48.9)
No	5	3	(60.0)	2	1	(50.0)	7	4	(57.1)
Has ways to release one’s stress									
Yes	57	30	(52.6)	31	11	(35.5)	88	41	(46.6)
No	4	3	(75.0)	3	3	(100.0)	7	6	(85.7)
Feels adequate reward for own work									
Yes	56	28	(50.0)	30	13	(43.3)	86	41	(47.7)
No	6	5	(83.3)	5	2	(40.0)	11	7	(63.6)
Work controllability									
Can	46	20	(43.5)	24	10	(41.7)	70	30	(42.9)
Cannot	16	13	(81.3)	11	5	(45.5)	27	18	(66.7)
SOC10									
≤ 45	32	20	(62.5)	16	10	(62.5)	48	30	(62.5)
> 45	30	13	(43.3)	19	5	(26.3)	49	18	(36.7)

Burnout: respondents who were diagnosed as burnout by the Maslach Burnout Inventory (emotional exhaustion ≥16 and/or cynicism ≥11)

n: number of respondents. SOC10: scores on the Sense of Coherence scale, excluding three items that were classified into the Maslach Burnout Inventory subscales

Logistic regression analysis of burnout using gender, frequency of night duty, working hours, age, work controllability, and the SOC10 as independent variables indicated that working hours (odds ratio [OR]: 1.315, 95% confidence interval [CI]: 1.057–1.636, *p* = 0.014) and the SOC10 (OR: 0.928, 95% CI: 0.882–0.976, *p* = 0.004) were significant factors for having burnout. The SOC10 (OR: 0.918, 95% CI: 0.854–0.986, *p* = 0.018) and frequency of night duty (OR: 0.615, 95% CI: 0.388–0.974, *p* = 0.038) were significant variables for burnout for respondents in the third and fifteenth months of the residency program, respectively.

### MBI subscale scores at different training phases

At the third month, 32 (51.6, 97.0% of burned out residents), 16 (25.8, 48.5% of burned out residents), and 47 (75.8%) residents were judged as having abnormally high scores on the MBI-EX and MBI-CY, and low scores on the MBI-PE, respectively (Table 5).

**Table 5:** Number of respondents who had abnormal MBI subscale scores in the third and fifteenth months of their residency program

	Third month		Fifteenth month		Total	
	n	(%)	n	(%)	n	(%)
High MBI-EX	32	(51.6)	12	(34.3)	44	(45.4)
High MBI-CY	16	(25.8)	9	(25.7)	25	(25.8)
Low MBI-PE	47	(75.8)	30	(85.7)	77	(79.4)
Burnout	33	(53.2)	15	(42.9)	48	(49.5)
Total	62	(100)	35	(100)	97	(100)

High MBI-EX: residents whose Maslach Burnout Inventory (MBI) emotional exhaustion score was ≥16

High MBI-CY: residents whose MBI cynicism score was ≥11

Low MBI-PE: residents whose MBI professional efficacy score was ≤25

Burnout: respondents who were diagnosed as having burnout by High MBI-EX and/or High MBI-CY

n: number of respondents

At the fifteenth month, 12 (34.3, 80.0% of burned out residents), 9 (25.7, 60.0% of burned out residents), and 30 (85.7%) residents were judged as having abnormally high scores on the MBI-EX and MBI-CY, and low scores on the MBI-PE, respectively.

Chi-square test and t-test indicated that the frequency of abnormal subscale scores and average subscale scores between the third and fifteenth months were not significantly different.

### MBI subscale scores, SOC10 score, and working hours

The average SOC10 score of the 97 residents was 45.0 (standard deviation 10.1) (Table 3). Thirty residents (62.5%) in the low SOC group (SOC10 ≤ 45, *n* = 48) and 18 (36.7%) in the high SOC group (SOC10 > 45, *n* = 49) were judged as having burnout (Table 4).

The MBI subscale scores in the low and high SOC groups in the third and fifteenth months are shown in Table 6. MBI-EX, −CY, and -PE scores between the low and high SOC groups at the third month, and MBI-CY, and -PE scores between the low and high SOC groups at the fifteenth month were significantly different by t-test (*p* < 0.05).

**Table 6:** Average MBI subscale scores in the low and high SOC groups and Pearson correlation coefficients with working hours

		Third month								Fifteenth month							
					*t*-test		Pearson correlation with working hours						*t*-test		Pearson correlation with working hours		
MBI	Group	n	Mean	SD	p (*d*)	1-β	r	p	1-β	n	Mean	SD	p (*d*)	1-β	r	p	1-β
-EX	Low SOC	32	17.7	6.8	0.048^*^ (0.51)	0.51	0.47	0.007^**^	0.79	16	15.9	6.8	0.083 (0.61)	0.41	0.17	0.525	0.15
	High SOC	30	14.2	6.9			0.28	0.134	0.32	19	11.7	6.8			0.17	0.479	0.15
-CY	Low SOC	32	9.9	7.2	0.001^**^ (0.89)	0.93	0.39	0.029^*^	0.61	16	12.9	8.6	0.000^**^ (1.31)	0.96	0.14	0.602	0.12
	High SOC	30	4.6	4.4			0.14	0.466	0.11	19	4.4	3.1			0.09	0.708	0.08
-PE	Low SOC	32	14.3	7.1	0.008^**^ (0.70)	0.78	−0.44	0.012^*^	0.73	16	12.9	6.4	0.049^*^ (0.70)	0.51	−0.19	0.474	0.18
	High SOC	30	19.7	8.1			0.36	0.049^*^	0.51	19	17.5	6.8			0.24	0.315	0.25

*MBI-EX* Maslach Burnout Inventory-emotional exhaustion

*MBI-CY* Maslach Burnout Inventory-cynicism

*MBI-PE* Maslach Burnout Inventory-professional efficacy

Low SOC: Respondents whose score on the 10-item Sense of Coherence Scale was ≤45

High SOC: Respondents whose score on the 10-item Sense of Coherence Scale was > 45

n: number of respondents. *SD* standard deviation

*p < 0.05; **p < 0.01: Significant difference in average MBI subscale scores between the low and high SOC groups based on a *t*-test, and in Pearson correlation analysis of MBI subscale scores and working hours

*d*:Cohen’s *d* effect size

1-β: Power of analysis

r: Pearson correlation coefficient

In the low SOC group, the Pearson correlation coefficients between working hours and all three MBI subscale scores were significant in the third month (MBI-EX: *r* = 0.47, *p* = 0.007; MBI-CY: *r* = 0.39, *p* = 0.029; MBI-PE: *r* = − 0.44, *p* = 0.012), but not in the fifteenth. In the high SOC group, working hours correlated with only MBI-PE in the third month (*r* = 0.36, *p* = 0.049) (Table 6).

To analyze the relationship between working hours, SOC scores, and MBI subscale scores, the respondents were divided into four groups: ≤9 h (19 residents), > 9 to 11 h (29 residents), > 11 to 13 h (32 residents), and > 13 h (17 residents) per day.

**Fig. 1:**
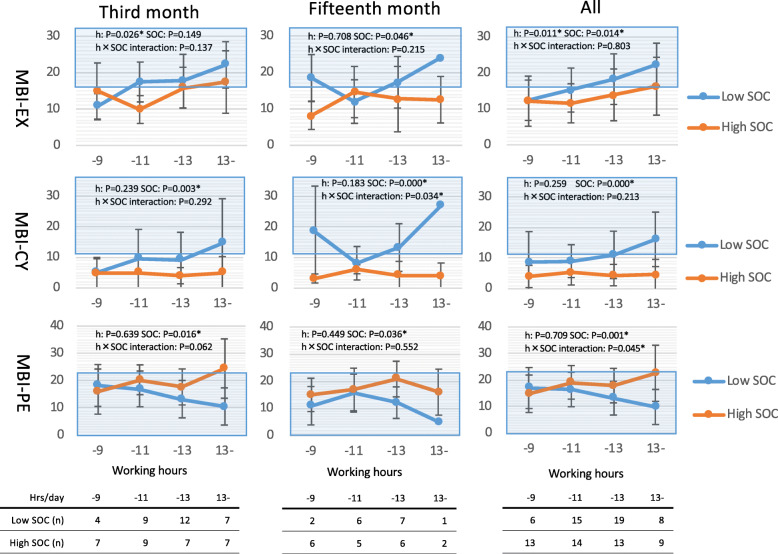
Average Maslach Burnout Inventory (MBI) subscale scores in the high and low Sense of Coherence (SOC) scale groups by working hours (− 9: ≤9 h/day; − 11: > 9 to 11 h/day; − 13: > 11 to 13 h/day; and 13–: > 13 h/day). MBI-EX: MBI-emotional exhaustion; MBI-CY:MBI-cynicism; MBI-PE:MBI-professional efficacy. The area with blue shadowing indicates clinical burnout or abnormally low professional efficacy. Low SOC: residents with a score of ≤45 on 10 items of the SOC scale (SOC10); High SOC: residents with a score > 45 on the SOC10. The significance of independent variables (h, SOC) and their interactions calculated by two-way analysis of variance for each MBI subscale score are indicated. *p < 0.05; **p < 0.01

As shown in Fig. 1, two-way analysis of variance with working hours and SOC scores as factors of the MBI-EX score revealed that working hours and SOC scores were significant variables in the third and fifteenth months, respectively. Regarding the MBI-CY and MBI-PE scores, SOC was a significant variable in both the third and fifteenth months. The MBI-PE score in the high SOC group was higher (more professional efficacy) in longer working hour groups, whereas the MBI-PE score of the low SOC group was lower (less professional efficacy) in longer working hour groups.

## Discussion

In the present study, 49.5% of Japanese residents were judged as having burnout, and working hours was significantly related to burnout. However, the residents in this study, who worked an average of 63.3 h per week, showed a high frequency of burnout similar to residents in the United States who worked an average of 80 h per week, which indicates that working hour regulations alone might not be adequate for reducing burnout.

The MBI-EX, which is composed of five items, e.g., ‘I feel emotionally drained from my work’, and ‘I feel tired when I get up in the morning and have to face another day on the job’, indicates decreased working vitality, that is, a low state of mental, creative, and physical energy levels. Meanwhile, the MBI-CY, which is composed of five items, e.g., ‘I’ve become less interested in my work since I started this job’, and ‘I doubt the significance of my work’, indicates decreased enthusiasm, that is, a low degree of interest in working. Our data indicated that half of Japanese residents were judged as having burnout as early as the third month of training, and among those burned out residents, 97.0% were emotionally exhausted and 48.4% were abnormally cynical.

Teunissen et al. pointed out a problem at the transitional phase from undergraduate education to postgraduate residency training [38]. Residents were under greater stresses caused by interacting with patients and medical staff, learning new material in a more self-directed way, and bearing many responsibilities that accompany the delivery of patient care and the need to take on increasingly more tasks independently [38]. Residents were mentally fatigued and had restrained emotions during this phase.

Burnout among Japanese medical students, whose clinical training is usually finished 8 months prior to graduation, was reported as being 13.3% for males and 31.3% for females [39]. When these students start residency training in a teaching hospital, there is a substantial change in their professional role as a licensed medical doctor and in their personal life. Encountering a new environment might be a cause of burnout among Japanese residents at the early phase of training, since working hours, one of the environmental factors, was a significant factor of MBI subscale scores at the third month of training.

Among burned out residents at the fifteenth month, 80% were emotionally exhausted, and 60% were abnormally cynical. At this point, they tended to have low enthusiasm and view things cynically in addition to having low interest in working compared to burned out residents at the third month.

Cynicism in the later phase of training has previously been reported [40], and our data indicate that residents with low stress coping ability might have this tendency.

Burnout as judged by the MBI indicates an individual’s state at the time of inquiry. On the other hand, a resident’s stress coping ability as evaluated by the SOC scale is fixed to some extent in early adulthood, and might be only slightly changeable because of life experiences [25]. As previously reported [23, 26], resident groups with high SOC scores showed a low frequency of burnout as judged by emotional exhaustion and cynicism. In addition, these residents showed high professional efficacy, regardless of the training phase. Furthermore, longer working hours did not worsen their emotional exhaustion or cynicism; rather, professional efficacy was increased in this group, in contrast to the low SOC group, in which professional efficacy decreased.

Kroninger-Jungaberle et al. reported that the concepts of SOC and self-efficacy foster resilience [41]. Mastery experience has also been reported to increase self-efficacy [42]. Our data indicate that residents with high stress coping ability can increase their self-efficacy by gaining more clinical experience, as indicated by working hours, thereby obtaining resilience.

A previous report pointed out that limiting working hours is obviously effective for the prevention of burnout, but there was some discussion that excessive restrictions might hinder professional development [29, 43]. We found that appropriate working hours for effective training might vary among individuals, and that in addition to supportive programs for residents, personalized programs compatible with his/her stress coping ability and current burnout status would be required.

Residents are in danger of burnout at the very early phase of training, and the expression of burnout might change as time passes. Screening residents using the SOC scale could help teaching staff and program directors identify residents at high risk of burnout and provide necessary support early for its prevention without excessively limiting clinical experience for those at low risk.

## Limitations

The hospital targeted in the present study was randomly selected from among those throughout the entire country, but the response rate and number of samples for analyses were low. We could not exclude the possibility that burned out residents did not respond to the questionnaire, or that the prevalence of burnout was higher than that identified in our analyses. In addition, we used a cross-sectional survey design with two data collection points. Thus, the relationship between working hours and burnout at a later phase of a training program remains somewhat unclear because long working hours could be a cause of burnout, and also shortened as a result of burnout. To confirm the changes in burnout status during the training period, it will be necessary to follow these individuals in a cohort study.

Furthermore, this was a survey of residents in a Japanese training program; additional research in other regions and with different training systems is required before our results can be generalized.

## Conclusions

Half of the Japanese residents analyzed in the present study were emotionally restrained and judged as having burnout in the third month of training. As previously reported, working hours and stress coping ability as evaluated by the SOC were both independent influential factors for burnout. In addition, we found that residents with high stress coping ability maintained their interest and enthusiasm for working and obtained professional efficacy under longer working hours, whereas residents with low stress coping ability were liable to experience burnout under the same working conditions. Individual stress coping ability could therefore be valuable information for fostering a suitable training environment.

## Data Availability

Data except MBI can be disclosed within the scope of ethical codes according to requests to Masami Tagawa.

## Abbreviations

MBI: Maslach Burnout Inventory
SOC: Sense of Coherence
MBI-GS: Maslach Burnout Inventory-General Survey
MBI-EX: MBI-GS subscale emotional exhaustion
MBI-CY: MBI-GS subscale cynicism
MBI-PE: MBI-GS subscale professional efficacy
OR: Odds ratio
CI: Confidence interval
